# A new predisposing factor for trigemino-cardiac reflex during subdural empyema drainage: a case report

**DOI:** 10.1186/1752-1947-4-391

**Published:** 2010-11-30

**Authors:** Toma Spiriev, Nora Sandu, Belachew Arasho, Slavomir Kondoff, Christo Tzekov, Bernhard Schaller

**Affiliations:** 1 Department of Neurosurgery, Tokuda Hospital, Sofia, Bulgaria; 2 Department of Neurosurgery, University Hospital Lariboisiere, Paris, France; 3 Department of Neurosurgery, University of Lausanne, Switzerland; 4 Department of Neurology, University Addis Ababa, Ethiopia

## Abstract

**Introduction:**

The trigemino-cardiac reflex is defined as the sudden onset of parasympathetic dysrhythmia, sympathetic hypotension, apnea, or gastric hypermotility during stimulation of any of the sensory branches of the trigeminal nerve. Clinically, trigemino-cardiac reflex has been reported to occur during neurosurgical skull-base surgery. Apart from the few clinical reports, the physiological function of this brainstem reflex has not yet been fully explored. Little is known regarding any predisposing factors related to the intraoperative occurrence of this reflex.

**Case presentation:**

We report the case of a 70-year-old Caucasian man who demonstrated a clearly expressed form of trigemino-cardiac reflex with severe bradycardia requiring intervention that was recorded during surgical removal of a large subdural empyema.

**Conclusion:**

To the best of our knowledge, this is the first report of an intracranial infection leading to perioperative trigemino-cardiac reflex. We therefore add a new predisposing factor for trigemino-cardiac reflex to the existing literature. Possible mechanisms are discussed in the light of the relevant literature.

## Introduction

For more than a century, it has been well known that electrical, chemical, or mechanical stimulation of the trigeminal nerve leads to trigemino-respiratory reflexes followed by cardiac arrhythmias [1]. In the early 20th century, this phenomenon gained increased clinical attention in the form of the oculocardiac reflex (OCR), which represents the cardiac response associated with stimulation of the ophthalmic division of the trigeminal nerve during ocular surgery [2]. In 1999, Schaller [3] demonstrated for the first time that a similar reflex occurs with stimulation of the intracranial (central) portion of the trigeminal nerve during skull-base surgery and subsummarized all these trigemino-depressor responses under the term "trigemino-cardiac reflex (TCR)" [4]. He also defined the TCR in a way that is now generally accepted. Later, his group also described the TCR for intraoperative stimulation of the peripheral portion [5].

Since then, there has been increasing discussion about the TCR itself, its provoking factors, and its treatment during intracranial or extracranial neurosurgical procedures. Several predisposing factors for intraoperative occurrence of TCR have been described [6-8], but until now no case of intracranial infection in combination with intraoperative TCR has been reported.

## Case presentation

### Preoperative history

A 70-year-old Caucasian man was admitted for the second time to the Department of Neurosurgery at our hospital. His personal history included symptomatic epilepsy and chronic anemia after nephrectomy because of kidney carcinoma two years before admission to our clinic.

**Figure 1:**
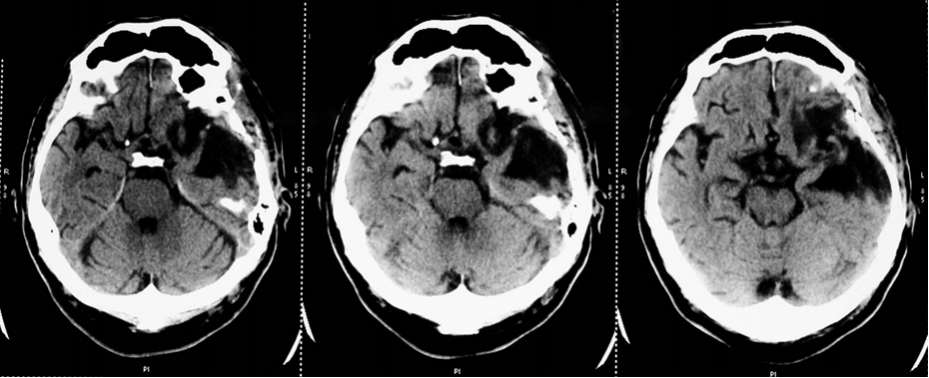
**Preoperative computed tomography (CT) scan**. Subdural collection with capsule organization and collateral brain edema on the side of the previous tumor is clearly visible.

**Figure 2:**
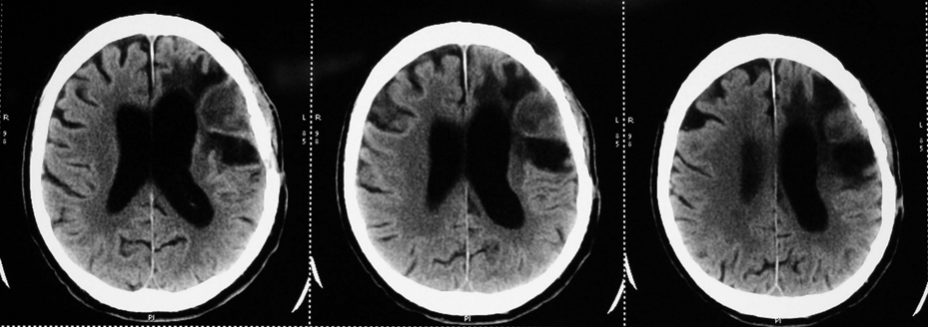
**Preoperative CT scan**. The surrounding tissues (including the dura) are thicker, related to the associated inflammation.

**Figure 3:**
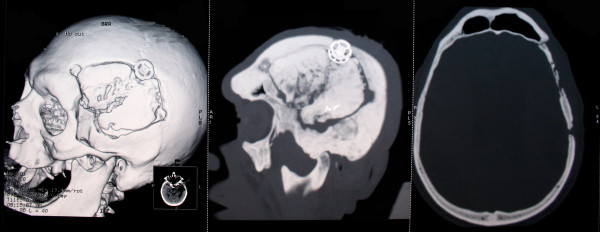
**Preoperative CT scan**. CT bone window shows partial osteolysis of the bone flap, due to osteomyelitic process.

Two months before the current admission, he underwent surgery for a giant left frontotemporal meningioma which was removed "gross totally." One month after this intervention, there was seen a fistula with emission of pus in the middle third of the operative scar. After another neurosurgical consultation, he was admitted to our department for surgery. At this occasion, the patient presented afebrile, with a blood pressure (BP) of 150/70 mmHg and a heart rate (HR) of 82 beats per minute (beats/minute), complaining of headache as well as vomiting. In the neurological examination, there was seen a right-side horizontal nystagmus, a right-side hemiparesis (MRC grade 3) and complete motor aphasia. The only medication that he was taking was carbamazepine 2× 200 mg for epileptic prophylaxis. On the cranial computed tomography (CT) scan without contrast performed in our hospital, a partial osteolysis of the frontotemporal bone flap was demonstrated, the surrounding tissues (including the dura) were seen as thicker (due to the associated inflammation), and a subdural collection with capsule organization and peri-lesional brain edema on the side of the previous tumor was described (see Figures 1, 2, and 3). On cranial CT bone reconstruction, the osteolytic foci and fistula were clearly visible. The laboratory examination showed, besides the chronic anemia, normal C-reactive protein but a monocytosis of 1.04 10^-9^/L (normal value, 0.1 to 0.8). The patient was diagnosed with a subdural empyema and an indication for the operative treatment was set.

### Anesthetic technique

The patient underwent surgery several days after this second hospitalization. No pre-operative antibiotics were given. The patient fasted for eight hours prior to surgery. Routine monitoring during surgery included electrocardiography (ECG), end-tidal (ET) concentration of CO_2 _and sevoflurane, and pulse oximetry. All hemodynamic parameters were monitored continuously and recorded throughout the neurosurgical procedure. Anesthesia was induced with midazolam (1 mg total dosage) and propofol (2 mg/kg) followed by suxamethonium chloride (1.1 mg/kg), atracurium (0.6 mg/kg), and fentanyl (100 μg total dosage). After the trachea was intubated, the lungs were mechanically ventilated (S/5 Aespire Config; Datex-Ohmeda Ins., Madison, WI, USA) with a mixture of air and O_2_. Anesthesia was maintained with sevoflurane (1%). An additional 50 mg of propofol and 1 mg of midazolam were applied during the intervention when necessary.

### Surgical technique and postoperative management

**Figure 4:**
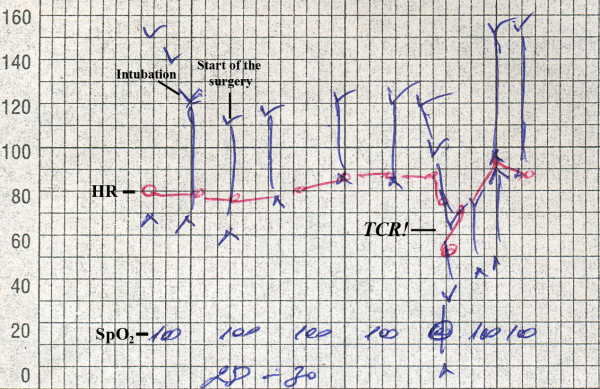
**Anesthesiology chart**. Before the occurrence of trigemino-cardiac reflex (TCR), mean arterial blood pressure (MABP) was 91.0 mmHg and heart rate (HR) was 82.5 beats/minute. At the time of the TCR record, the patient's blood pressure dropped to 37/0 mmHg (MABP, 12.3 mmHg; 86.49% drop from baseline), and concomitantly HR dropped to 61 beats/minute (26.07% drop from baseline). No significant blood loss at the time of the incident was recorded. The applied medications were ephedrin (20 mg), atropin (0.5 mg) and methylprednisolone (60 mg). After drug administration, the patient's hemodynamic parameters returned to normal and the intervention was reinitiated.

A frontotemporal skin incision was made using the same method used in the first intervention. Between the bone flap and galea aponeurotica in the left frontotemporal region, a large quantity (approximately 7-12 ml) of pus was removed. Intraoperatively, the bone flap was found to be changed by the osteomyelitic process. It was eroded by the inflammation, with multiple pus-filled channels connecting the inner and outer bone tables. After opening the dura, a gray-white thick pus was removed. During the whole intervention, the patient's baseline mean arterial blood pressure (MABP) was 91.0 mmHg (range, 76.7-98.7 mmHg), and baseline mean heart rate (HR) was 82.5 bpm (range, 80-89 bpm). One hour and 20 minutes after skin incision during the removal of subdural pus and working around the dura, the patient's blood pressure dropped to 37/0 mmHg (MABP, 12.3 mmHg; a 86.49% drop from baseline) and concomitantly HR dropped to 61 bpm (a 26.07% drop from baseline). There was no significant blood loss at the time of the incident. The surgical procedure was discontinued, and the patient was given ephedrin (20 mg), atropin (0.5 mg), and methylprednisolone (60 mg) (see Figure 4). Two to three minutes after the administration of these drugs, the patient's hemodynamic parameters returned to normal, and the surgical intervention was continued. This phenomenon was reproducible. The skin fistulae were excised, and two subgaleal drainage systems (Dainobag Lock 300 V; B. Braun, Melsungen, Germany) with a diameter of 12 mm were left. The patient's postoperative period was uneventful, and he presented with no additional neurological deficit. On microbiological examination, actinomycosis was reported as the cause of the empyema that was treated with cefoperazone 2× 1 g for 12 days. The patient's C-reactive protein and leucocyte count remained normal. The postoperative period was uneventful. The patient was discharged from our hospital 13 days after the intervention.

## Discussion

The presented case report is unique and adds a new and important risk factor for the intraoperative occurrence of TCR to the existing literature. It seems that infected intracranial tissue may be a new predisposing factor in combination with surgical manipulation on the meninges, a routine surgical operative technique that has never been described before to be associated with TCR occurrence.

It has already been shown that mechanical stimulation of the cerebral falx results in hyperactivity of trigeminal ganglion, thereby triggering the TCR [9]. The neural supply of the cranial dura mater involves mainly the three divisions of the trigeminal nerve, the first three cervical spinal nerves, and the cervical sympathetic trunk. A case of immediate, reproducible, and reflexive response of asystole upon stimulation of the cerebral falx during operative resection of a parafalcine meningioma was previously reported [9], being most likely related to bilateral trigeminal stimulation of the falx. According to the studies of Penfield and McNaughton [10], the nervus tentorii, a recurrent branch of the ophthalmic branch of the trigeminal nerve bilaterally innervates the tentorium cerebelli, the dura of the parieto-occipital region, the posterior third of the falx, and the adjacent sinuses. In our present case, however, the subdural empyema was located in the middle cranial fossa that is predominantly innervated by the V2 and V3 branches of trigeminal nerve [11]. However, it has been previously shown by us and others that surgical procedures at the anterior, middle, and posterior skull base (any branch of the central part of trigeminal nerve) may elicit the TCR.

In this special case, one may suggest that the patient had simply a (physiological) Cushing reflex with consecutive elevated MABP before operation that only normalized after elevation of the mass lesion. But the Cushing reflex is not a possible explanation of the MABP and HF drop as seen in our case. In our case, the intraoperative phenomenon was reproducible, which would be not the case if there were a Cushing reflex. Our case shows, therefore, a clear cause-and-effect relationship necessary for the TCR and as described earlier in detail [3].

Different retrospective studies have shown an incidence of TCR ranging from 8% [12] to 18% [13] using all the same inclusion criteria as defined earlier by us [3]. However, it seems that TCR is often unrecognized intraoperatively, so the identification of possible provoking factors is important but often elusive. There are several reports for the provoking factor for the peripheral initiation and central initiation of the TCR. To date, several risk factors for the intraoperative occurrence of TCR have been identified, such as light general anesthesia, childhood, and the nature of the provoking stimulus (strength and duration of stimulus) [3,8]. In addition, there are several known provoking drugs such as potent narcotic agents (sufentanil and alfentanil), β-blockers, and calcium channel blockers [3,8]. Until now, no report for intracranial infections as a provoking factor for intraoperative TCR occurrence has been identified.

Intracranial infections, as in the current case of subdural empyema, could lead to a pathological process called sensitization of trigeminal afferents in the dura mater [14]. It was demonstrated that chemical stimulation of dural receptive fields with inflammatory mediators such as prostaglandin E_2_, bradykinin, or histamine directly excite the neurons and enhance their mechanical sensitivity [1,5], such that they can be easily activated by mechanical stimuli that initially had evoked little or no response [14,15]. It seems that meningeal sensory innervation is not known to subserve multiple sensory modalities [10,14]. Meningeal afferents are thought to become activated only under potentially harmful or pathological conditions [10]. However, although the dural afferent population does not appear to mediate distinct sensory modalities, it shows a pattern of variation in mechanosensitivity as a function of conduction velocities [10,16]. Mechanical response properties of dura are attributed to A and C primary afferent neurons. Such exaggerated mechanical sensitivity and manipulation of the dura mater could play a role in the initiation of TCR in our case.

## Conclusion

To the best of our knowledge, this is the first report of an intracranial infection with the intra-operative occurrence of TCR during a routine neurosurgical maneuver. Infected (intracranial) tissue may be a new and important predisposing factor for the occurrence of TCR, a phenomenon that is different from the falcine TCR caused by bilateral stimulation of tentorial nerve that was described earlier. Further laboratory and clinical investigations are needed to clarify this new information about TCR.

## Competing interests

The authors declare that they have no competing interests.

## Consent

Written informed consent was obtained form the patient for publication of this case report and accompanying images. A copy of the written consent is available for review by the Editor-in-chief of this journal.

## Authors' contributions

TS and BS wrote the article. TS collected the data. BS interpreted and analyzed the data. SK and CK performed the operation and the patient's treatment and provided substantial information regarding the patient's case and were therefore major contributors to writing the manuscripts. NS and BA provided some specific and general ideas that initiated the work and helped to finish the work. Without both contributions, this report would not have been possible. NS made substantial corrections to the manuscript. All authors read and approved the final manuscript.
